# Entropy is a Simple Measure of the Antibody Profile and is an Indicator of Health Status: A Proof of Concept

**DOI:** 10.1038/s41598-017-18469-6

**Published:** 2017-12-22

**Authors:** Lu Wang, Kurt Whittemore, Stephen Albert Johnston, Phillip Stafford

**Affiliations:** 1 Center for Innovations in Medicine, Biodesign Institute, Arizona State University, Tempe, AZ 85287 United States; 2 Centro Nacional de Investigaciones Oncologicas, Madrid, 28029 Spain

**Keywords:** Peptides, Applied immunology

## Abstract

We have previously shown that the diversity of antibodies in an individual can be displayed on chips on which 130,000 peptides chosen from random sequence space have been synthesized. This immunosignature technology is unbiased in displaying antibody diversity relative to natural sequence space, and has been shown to have diagnostic and prognostic potential for a wide variety of diseases and vaccines. Here we show that a global measure such as Shannon’s entropy can be calculated for each immunosignature. The immune entropy was measured across a diverse set of 800 people and in 5 individuals over 3 months. The immune entropy is affected by some population characteristics and varies widely across individuals. We find that people with infections or breast cancer, generally have higher entropy values than non-diseased individuals. We propose that the immune entropy as measured from immunosignatures may be a simple method to monitor health in individuals and populations.

## Introduction

The antibodies in an individual’s blood offer a tremendously valuable source of information. The 10^9^ types in an individual and 10^12^ total variants exist in widely different concentrations and affinities for their original targets^1^. There are also 5 major isotypes adding to the richness of this information^2^. Many strategies have been employed to decipher this complexity. Arrays of proteins representing some or all of the proteome of a species are produced commercially^3–6^. These can be used to discover antibodies against pathogen proteins or autoantibodies. Peptide arrays representing the proteomes provide higher resolution for the antibody binding to known proteins. Alternatively, high throughput sequencing can be used to read the total variable regions of B and T cells^7,8^. The composite of all of the sequences represents the profile of the antibody coding regions for a particular sample. We have developed an approach, immunosignatures (IMS) that also uses peptide arrays, but the peptides are chosen from random sequence space to maximize chemical diversity and to allow for the presence of mimotopes to epitopes which may be novel, such as a mutation in a cancer cell^9,10^. These peptide arrays can be used to discover biomarkers or vaccine candidates^1,11–13^. IMS can be used as a diagnostic tool^1,12,14–16^. In contrast, here we use explore the application of IMS to measure the immune entropy of individuals across time, populations and health status.

The IMS technology is based on creating arrays of 10^4^ to 3 × 10^5^ peptides, 9–20 amino acids long, in an area of ~0.5 cm^2 ^^9,14,17,18^. They are chosen from random peptide sequence space to optimize chemical diversity and therefore, presumably, binding distinctions between antibodies. Given that most epitopes of antibodies are 5–20aa long, it is unlikely that the exact cognate epitope for any antibody is present in the arrays. However, because of the avidity effect each antibody will bind many peptides in a characteristic signature^10,18^. Therefore, when blood from an individual is applied, a complex pattern of antibody binding (immunosignature, IMS) is produced unique for each sample. The binding varies in which features are bound and the amount of antibody on each feature. An attractive feature of IMS is its simplicity. A drop of blood can be sent on a filter paper thru the mail, diluted and applied to the array to make the measurement, greatly facilitating monitoring individuals^19^.

Here we calculate the information entropy of each IMS. Shannon information entropy (defined as H = −∑ p(x) * log(p(x)) where p(x) is the probability of outcome x) can be applied to any type of information to quantify how predictable the information is. In information theory, the entropy can be determined from the frequency of values for all of the elements contained in an object of information. For example, the entropy of the message “aaaa” would have a lower entropy value than the message “abcd”. The entropy value of the first message is −(4/4 * log(4/4)) = 0, and the entropy of the second message is −(1/4 * log(1/4) + 1/4 * log(1/4) + 1/4 * log(1/4) + 1/4 * log(1/4)) = 1.39. Therefore, high entropy information is most similar to the information that would be output by a random information generator.

Global measures, and the entropy measure in particular, have been applied to a variety of biological data previously. Global measures such as the mean and median of a sample are used extensively in scientific research. Application of information entropy is less common, but it has been used to characterize a wide range of different biological data. In cancer, the entropy calculated from aberrations in DNA copy number is higher in a variety of cancer types^20^, alternative splicing entropy is higher in some cancers^21^, the entropy of structural and numerical chromosomal aberrations is higher in cancers^22^, the entropy of a random walk on the protein interaction network graph was higher in cancer cells^23^, and the entropy of photographs of tissues was higher in cancer tissues^24^. In the brain, the entropy of fMRI data increases with age and Alzheimer’s disease in a dataset of 1,248 samples^25,26^. Schizophrenic patients had a lower entropy value than normal subjects, which indicates that entropy values that are too low or too high may indicate that something is altered from normal in the system being investigated^25^. Rhesus monkeys with induced Parkinson’s disease had higher levels of neuronal firing entropy compared to controls^27^. Entropy has also been used for data related to the immune system. For example, Vilar *et al*. assessed entropy from data sets on immune cells^28^. Merilli *et al*. applied entropy values to the putative idiotypic network of antibodies^29^. Asti *et al*. used maximum-entropy models based on antibody gene sequence data to predict antibody binding from complex mixtures^30^.

Here we calculate the Shannon information entropy of the peptide fluorescence intensity distribution that results from applying sera to a complex peptide microarray surface. The immune entropy (IE) was measured in a wide array of people, the same people over time and the people with diseases.

## Results

### Entropy can differentiate a monoclonal antibody solution from a mixed antibody solution

**Figure 1:**
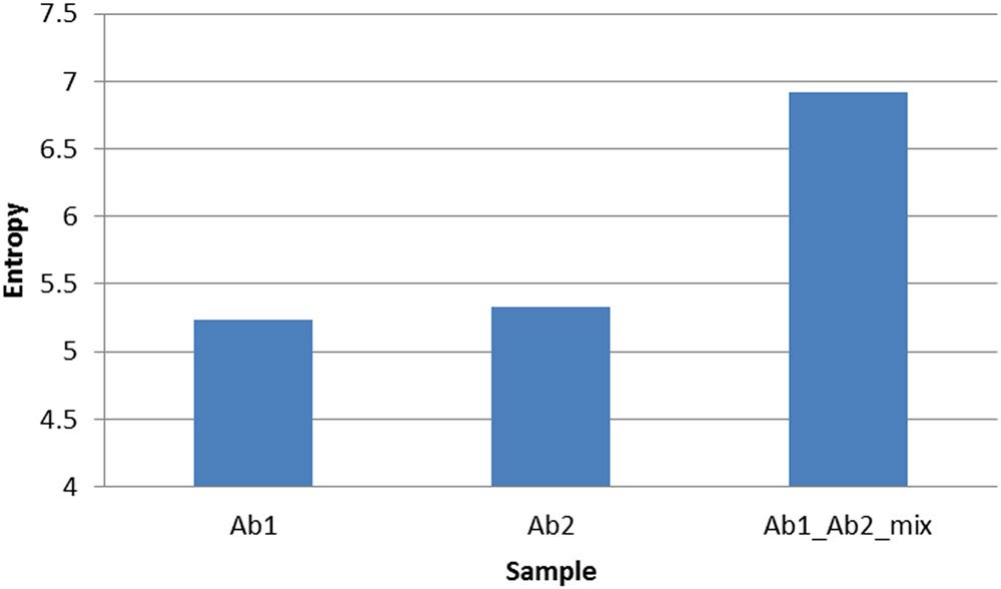
Entropy measurement is able to distinguish a single monoclonal antibody profile from a mixed monoclonal profile. Antibody1 and antibody 2 are individually applied to the Immunosignature platform and then mixed together to apply for the Immunosignature platform. The entropy value is calculated for each distribution. The two monoclonal antibody entropies cannot be differentiated, while both of them are obviously lower than mixing the two antibodies together.

Entropy can generally measure the difference in the distribution of two datasets as illustrated by example in Figure S1. As applied to an IMS, the expectation is that more antibody types would produce more randomness, which should result in a higher entropy number. This hypothesis was tested by measuring the entropy of binding of two different monoclonal antibodies individually and then in an equal mixture. The results are shown in Fig. 1. The two monoclonals target different sites (RHSVV and SDLWKL) on the p53 protein. When each was applied separately to the array, they bound a different set of peptides but the distribution was approximately the same, so the IEs were similar. However, when the two antibodies were mixed, the distribution of the IMS signal expanded, which in turn caused the entropy to be higher than a single antibody. This result confirms that entropy can in principle be used as a measure of the disorder in an IMS.

### IE varies with gender, blood type, and ethnicity but not age or location

In order to identify factors associated with IE, we examined the sera of 800 healthy individuals using the IMS platform. These samples were obtained from Clinical Testing Solutions (CTS Inc., Tempe, AZ) and were chosen to equally represent the proportion of genders, ethnicity, blood types, and ages in the Southwest US population. They were collected from centers in California, Arizona and Texas.

**Figure 2:**
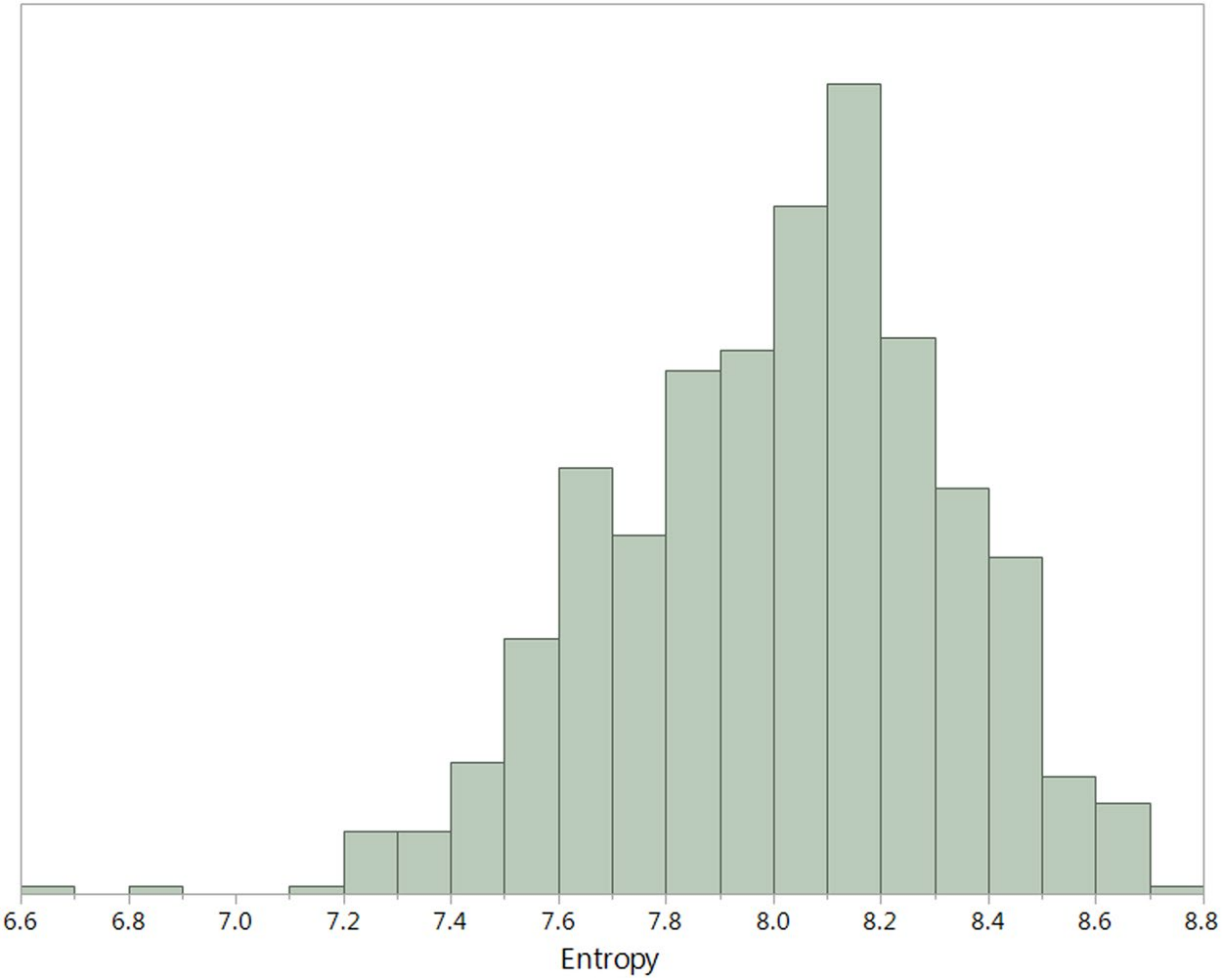
Distribution of entropy values for 800 healthy individuals. The entropy value ranges from 6.6 to 8.8 with a median of 8.1. The distribution is approximately normal.

In Fig. 2 the distribution of entropy values across the whole set of 800 samples is presented. The entropy values ranged from 6.6 to 8.8 with a median of 8.1. The values are approximately normally distributed. There was no significant difference from a normal distribution in a Normal Quantile Plot. Applying non-parametric tests for the same data were also significantly different.

**Figure 3:**
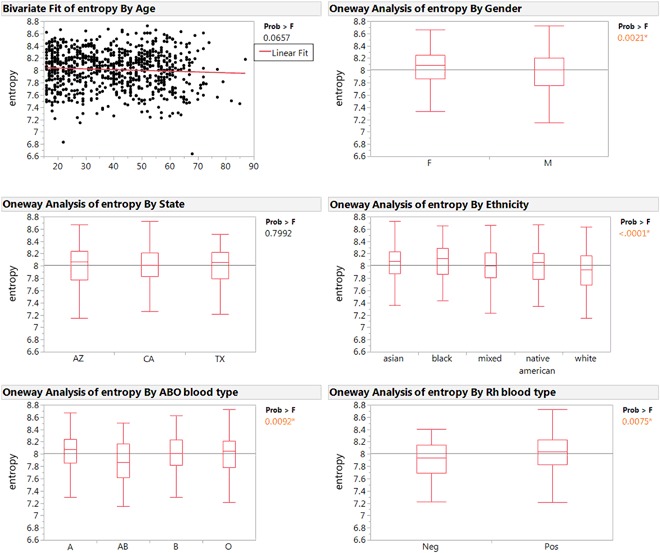
Entropy measurement variance by different factors. Entropy value was tested with factors of age, gender, location (state), ethnicity and blood type. Age, gender, and location are found to not influence the entropy value, while ethnicity and blood type has significant influence on the entropy value. The p-value is obtained from an ANOVA test for each comparison.

Figure 3 shows the IE distribution with various factors including age, location, gender, blood type, and ethnicity. The distribution in every group follows a near normal distribution. We asked if there were any significant differences in pairwise comparisons of the entropy with regard to these factors. We found none with respect to age and location. However, we did find that the entropy values are influenced by gender, blood type, and ethnicity.

Generally, females have slightly higher entropy than males. Caucasians had a lower entropy level than Asian or African-Americans. The difference of these two sets of comparisons were at a significance level of <0.005 by a t-Test and <0.0001 by an ANOVA test.

We found differences in IE both in the ABO blood group system and the Rh blood group system. People with AB blood type have on average the lowest entropy value, whereas the other blood types are similar to each other. The Rh blood system also shows that Rh- blood type has lower entropy compared with Rh + blood type.

As noted the Caucasian and Asian populations had different average entropy levels and Rh + and Rh- have different average values. Caucasians have a frequency of 17% for Rh- while Asians have a frequency of <2%^31^. Given these differences we inquired whether the differences in ethnic backgrounds could be accounted for by Rh differences. The Rh- samples were subtracted from the Asian and Caucasian derived samples and reanalyzed. The difference in entropy averages was not affected. Therefore, it appears the differences at least between the Asian and Caucasian groups is not due to differences in Rh factor.

### The entropy value varies between individuals, in the same individual over time, and can reflect health status

**Figure 4:**
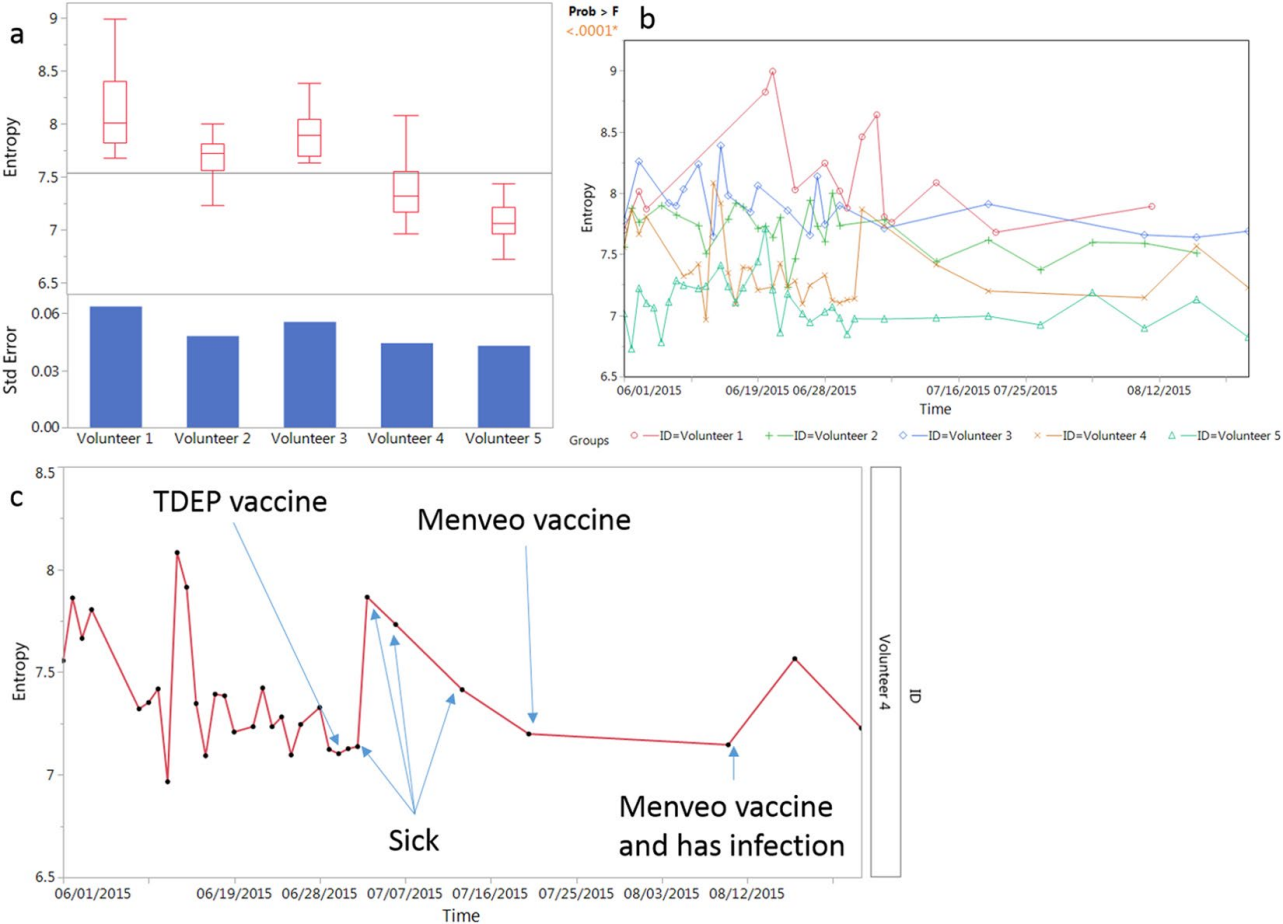
Entropy measurement variance between individuals over time and with changes in health states. (**a**) boxplot of 5 individual’s entropy recorded over a period of time shows difference from person to person. (**b**) plotting entropy against time for the volunteers shows variation of entropy that is independent between individuals. (**c**) Recorded volunteer’s activity shows entropy changes with vaccine administration and sickness. Black dots are blood draw points and the red line connects the dots.

One would assume that the entropy value between individuals would be different even if just due to random fluctuations in the immune system. However, it is not known what the range of the variation is and how it differs from person to person. In this experiment, we obtained the IMS of 5 individuals over a period of time. Blood was drawn daily for 1 month and every week for 2 subsequent months, the IMS determined for each sample and the entropy calculated. The variance for each individual is summarized in a box plot in Fig. 4a. An ANOVA test shows a p-value < 0.0001, indicating there is significant difference from the grand mean in the mean entropy for the five individuals. This suggests that random fluctuations alone are not sufficient to explain the difference between individuals. It is of interest to note that people with lower average entropy tend to have lower variation overall. The standard error correlates well with the average entropy value. This is especially the case for volunteers 4 and 5, both of whom had the lowest average entropy and variance.

We were also interested in how entropy changes over time within an individual and between them. Instead of plotting the entropy values in a boxplot graph, we illustrated the entropy change with time in each of the individuals in Fig. 4b. Five volunteers are monitored during the same time period. As it shown, the entropy for all individuals varies during this period and does not show a time correlation between individuals. It appears that the variance in entropy is quite different between individuals.

To determine whether entropy can truly reflect the health status of an individual, we recorded the volunteers’ health and vaccine history during the monitored time period. An example of one individual is graphed in Fig. 4c. Volunteer 4 received 3 vaccines, and was self-reported sick during the monitoring period. Aside from the missing data points from July 25^th^ to early August, we found that there was a trend for the entropy value to increase on health intervention. This gives us a first indication that entropy can be used to monitor health status as it changes with exposure to infections or vaccines.

### Entropy is higher for people infected with pathogens

Once we established how entropy changes in healthy individuals, we asked whether entropy value changes with different forms of health disturbance. We first tested this with infectious diseases. Sera from 7 types of infections were assayed, including Borrelia (8), Bordetella pertussis (12), dengue (9), Hepatitis B virus (15), malaria (13), syphilis (8) and West Nile Virus (21). All samples were from convalescent people. These pathogens, including bacterial, viral and parasite infections, were chosen to broadly reflect the infectious population.

**Figure 5:**
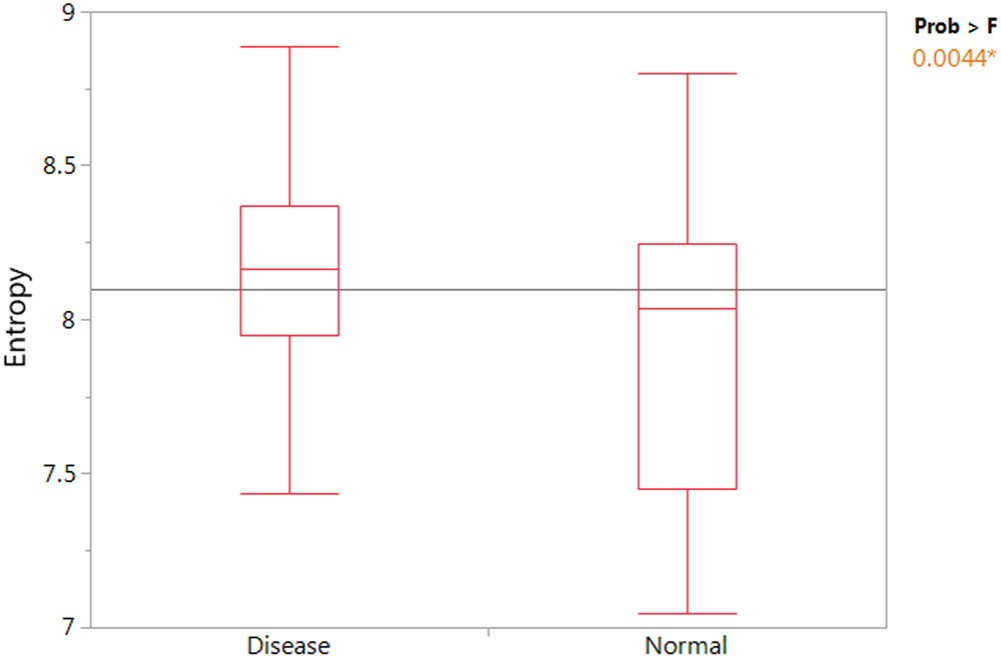
People recovering from infectious diseases have a higher entropy values compared with normal donors. Samples from 7 types of infections are mixed together to represent the disease group. A t-test shows that the entropy from the disease group is significantly higher compared with the normal donors. P-value < 0.0044.

When comparing them with non-infected samples, the infection group shows significantly higher entropy level (Fig. 5). This result implies that entropy can indeed distinguish people with different health status. Result of the un-mixed 7pathogens’ entropy comparison is attached in Figure S2.

### Sera from people with cancer exhibited a higher level of entropy

We also tested if people with cancer have differences in average entropy. Cancer signatures are distinct by type and from infections^32,33^. A tumor presumably presents more antigens, including neo-antigens, to the immune system and is often subject to immune suppression^34–37^.

**Figure 6:**
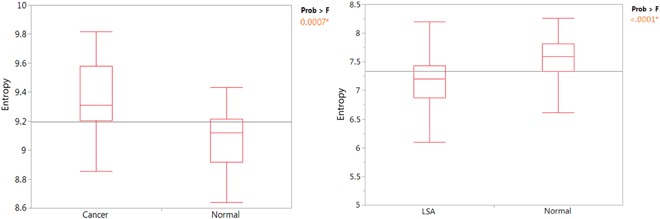
Comparision of cancer patients with normal donors. (**a**) Various cancer samples are used to represent the general cancer group. The boxplot shows that cancer samples have a higher entropy value compared with normal donors by T-Test with p-value < 0.0096. (**b**) Dog LSA samples are compared with non-cancer normal samples shows lowered entropy for LSA samples with p-value < 0.0001 by T-Test.

Here we used datasets from normal donors and from people with several types of cancer to represent general cancer patients, including breast cancer (5), esophageal cancer (2), *Glioblastoma multiforme* (1), lung cancer (1), meningioma (1) and multiple myeloma (1). Analysis is performed with sample sizes of 11 cancer and 21 healthy donors. As shown in Fig. 6a, cancer samples have significantly higher entropy value compared with healthy donors. The P-value from T-Tests is <0.0096.

In some B-cell lymphomas, a large amount of the same antibody is produced, which changes the antibody composition in the blood^38,39^. We predict that this may lead to lower entropy value compared with healthy donors. To test this prediction we determined the IMS for dogs with a B-cell lymphosarcoma (LSA) to healthy dogs. IMS uses the same chip for all diseases and species, just requiring the appropriate, in this case dog, secondary, labeled antibody. 68 normal dogs were compared to 83 LSA samples. As evident the entropy is significantly lower in the LSA compared with healthy dogs. This is consistent with the prediction.

## Discussion

We have explored the application of Shannon information entropy to immunosignatures. We first showed that two different monoclonal antibodies that bind to a different set of peptides and have comparable entropy measures, produce an increase in entropy when mixed and added to the arrays, as predicted. We then used a collection of sera from 800 people who equally represent gender, age, ethnic background and three geographic locations to measure the entropy of IMS for each. We found that the entropy values ranged from ~6.6 to 8.8 and were approximately normally distributed over the 800 samples. In pairwise comparison of various sets of signatures we found that there was no significant differences in average entropy values between age or geographic location. We did find the average values females were slightly higher than males, and Asian and African-American donors were significantly higher than that of Caucasian donors. While there were no differences in averages between A, B and O blood types, AB blood types were significantly lower on average. Rh- samples were on average lower than Rh+ . We found that the difference between Asian and Caucasian donor samples could not be explained by differences on Rh- frequency between the two groups. We extended the analysis to samples from people infected with 7 different pathogens and found that as a pool these samples had on average significantly higher entropy values than uninfected controls. The same was true for samples from people with three different cancers compared to people without cancer. However, we found that dogs with a B-cell lymphoma, as might be predicted for a clonal production of a particular antibody, actually had lower average entropy levels.

In the proof of principle experiment we used two different high affinity monoclonal antibodies to two different sites on P53 (Fig. 1). We have shown that monoclonal antibodies can vary greatly in the number of peptides they bind in the array^10^. We suggest that the entropy assessment of an antibody may be a good predictor of off-target binding. It would have the value of being a simple, single number standard that could be applied to all antibodies.

While there was a wide range of entropy values in each of the groups in the 800 samples (Fig. 2), there were significant differences in the average for gender, ethnicity, and blood groups (Fig. 3). The underlying causes of these differences is unknown. Given that the immune system is highly sensitive to both intrinsic and extrinsic factors it would take more studies to associated a cause(s) of the differences. Where there are no significant differences, for example geographic location, we can exclude differences in flora, for example, as inducing different average entropy levels.

Five people were monitored daily for one month and then weekly for an addition two months (Fig. 4). This allowed us to determine the differences in averages overtime and the variance for each person over time. The entropy averages of the 5 people happened to represent approximately the range we observed in the 800 samples. Each person generally maintained the differences between each other over the three months. The person with the highest average entropy also had the highest variance and the one with the lowest the lowest variance. It will be interesting to see in a larger set of individuals whether this generally holds true. In order to see if a health event changed the entropy value of an individual, one person received a vaccine. There was subsequently a sharp increase in the entropy number for this individual (Fig. 4c), although the increase was within the range they previously presented. Additionally, one individual later had an undiagnosed illness and this was accompanied by an increase in entropy (Figure S2). These are single events so the association between entropy increase and illness could be coincidental.

The results of the monitoring of individuals suggests two potential applications for entropy monitoring. On an individual level if a person monitors their entropy over time on a regular basis, one could detect a significant change from baseline or normal variance. To be useful this would change would need to be present before symptoms occurred. Whether entropy changes are present before symptoms is another area of future investigation.

Another potential application would be for population monitoring for a disease outbreak or an intentional biological attack. If a population was monitoring their IMS on a regular basis, presumably in order to detect early signs of a chronic disease, a disturbance in the entropy levels of a large number of people could be an indicator of an event. As evident from the data in Fig. 4 on monitoring individuals, this would need to be based on multiple measures of time of each individual. It may be possible to identify the peptides that were responsible for the change in entropy in each person and determine if there was a common signature. In the case of a natural outbreak or attack, this signature would represent the immune response to the infectious agent.

In the data presented in Figs 4c and S2, the disturbance health event was accompanied by an increase in entropy. We investigated whether this is generally the case. We found that for both infections (Fig. 5) and cancers (Fig. 6a) the people with the health problem had on average higher entropy levels. However, within both diseases there was a wide range in entropy values for different people. Therefore, even for a health disturbance that causes and increase in entropy, it would need to be measured against the personal baseline. As an example of entropy decreasing we presented analysis of dogs diagnosed with a B-cell lymphosarcoma (LSA). In contrast to the data in Fig. 6a, the average entropy was lower in the disease state. B-cell cancers may be a special case as they are characterized by overproduction of one antibody species.

Infections induce a set of high affinity antibodies to the pathogen. In order for this to register as an increase in entropy the induced antibodies would need to expand the number of sites bound relative to the peptides bound by the non-infected samples. The implication is that there would need to be unoccupied features that the induced antibodies could bind to expand the diversity. Presumably, this would also be the case for the cancer samples. In the case of the LSA samples the preponderance of the antibody produced by the cancerous B-cell would decrease the total diversity of antibodies in the sample to lead to a decrease in average entropy.

As discussed in the Introduction, the concept of entropy has been applied to various measures of the immune system. The approach of sequencing B-cell variable regions in depth most closely resembles our concept. For example, Asti *et al*.^28^ used deep sequencing data on HIV patients as applied to predict binding to HIV antigens. Using IMS to measure entropy of the antibody repertoire has several advantages. The blood spots for the IMS analysis can be sent through regular mail and only requires a small amount of blood, making large population surveys feasible^19^. The assay itself is simple and inexpensive. We hope that the simplicity of this approach to measuring the humoral immune component will encourage further investigations and applications.

## Material and Methods

### Array Platforms

Two different immunosignature peptide array platforms were used: two different libraries of 10,000 peptide microarrays, the CIM10Kv1(NCBI GEO accession number pending), the CIM10Kv2 (GPL17600) and HT330K (GPL17679). The 10 K random peptide platforms consists of 10 K 20 residue peptides linked to glass slides through a maleimide conjugation to a linker coupled to an aminosilane-coated glass surface. This linker is on the carboxyl terminus for CIM10Kv1 and on the amino terminus for CIM10Kv2^18^. The CIM10Kv1 arrays were produced by spotting peptides synthesized by Alta Biosciences using a NanoPrint LM60 microarray printer (Arrayit, Sunnyvale, CA). The CIM10Kv2, peptides were synthesized by Sigma Genosys (St. Louis, MO), and they were printed by Applied Microarrays (Tempe, AZ) using a piezo non-contact printer.

The 330 K platform (GPL17679) uses an *in situ* synthesis method to create 330,000 peptides on a silicon wafer^40^. This platform uses peptides selected from random space to maximally distribute the peptides in that space. On this platform, not all of the peptides have exactly the same length, but average 12 amino acids plus or minus 6 amino acids at the 95^th^ percentile. Arrays are deprotected following synthesis, soaked overnight in dimethyl formamide. The residual DMF was removed by two 5 min washes in distilled water, then arrays are soaked in PBS pH 7.3 for 30 min, blocked with an incubation buffer (3% BSA in Phosphate Buffered Saline, 0.05% Tween 20 (PBST)), washed, and spun dry, 1500RPM × 5′. At this point the, the arrays were ready for the application of sera.

### Array procedures with samples

The general assay conditions have been published previously^10,41–43^, and briefly described here. The procedure for applying sample to the arrays of the two different types of platforms is nearly identical, and less than 1 µl of sample is required. For the CIM10K platform, the microarrays are pre-washed in 10% acetonitrile, 1% BSA to remove unbound peptides. Then the slides are blocked with 1XPBS pH 7.3, 3% BSA, 0.05% Tween 20, 0.014% β-mercaptohexanol for 1 hr RT. Without drying, slides are immersed in sample buffer consisting of 3% BSA, 1X PBS, and 0.05% Tween 20 pH 7.2. Serum is diluted 1:500 and applied to the peptide array for 1 hr at 37 °C. The slides are washed in 1X Tris-buffered saline with 0.05% Tween 20 (TBST) pH 7.2. Then a mouse anti-human secondary antibody conjugated to a dye is applied to the array. The slides are washed again as before and dried by centrifugation. The slides are then scanned in an Agilent ‘C’ scanner to determine the intensity of each peptide. For the 330 k platform, the arrays were loaded into a multi-well Array-It gasket. Then a volume of 100 µl of incubation buffer was added to each well, and then 100 µl of 1:2,500 diluted sera was added for a final concentration of 1:5,000. Arrays were incubated for 1 hr at room temperature (RT) with rocking, and then washed with PBST using a BioTek 405TS plate washer. An anti-human IgG-DyLight 549 secondary antibody with a conjugated dye (KPL, Gaithersburg, MD) was added to the sera at a final concentration of 5 nM. This solution was incubated 1 hr at RT with rocking, and unbound secondary was then removed with PBST followed by distilled water. The arrays were removed from the gasket while submerged, dunked in isopropanol, and centrifuged dry at 800 × g for 5 min. These arrays were then scanned with a commercially available scanner to determine the intensity of a certain wavelength at each peptide feature position.

Once the 16 bit TIFF image file from either type of array was obtained, the intensity values from each feature were obtained using GenePix 8.0 (Molecular Devices, Santa Clara, CA). These fluorescence intensity values were then used to calculate the value of global measures such as the mean and Shannon information entropy.

### Java Entropy program

A custom Java program was written to calculate Shannon’s entropy from the fluorescence intensity files (.gpr, or “Gene Pix Array Format”) from the peptide microarray. Most image alignment software allows output as a gpr file, and that is how the program recognizes data columns. However, any datatype could be used with minor modifications. There are two programs listed in the Appendix, an algorithm class and a test class. The algorithm class provides values entropy given an immunosignature data file, but for comparison sake it also provides CV (coefficient of variance), mean, median, kurtosis, skew, 95^th^ percentile, 5^th^ percentile, and dynamic range. Tests have shown that entropy is the most sensitive and robust to health changes, but the other calculations provide comparisons. The test class allows the user to input their data directories and filenames, and serves as the Java main class.

### Software and statistics for general analysis

Microsoft Excel and JMP were used for data analysis and to create the graphs. Linear fit of entropy on age is by ordinary least squares. P-value is the probability of aging is actually influencing entropy. Either ANOVA test or t-Test is used in testing if entropy is being influenced by specific factors.

## Electronic supplementary material

Supplemental information
