# Author Correction: Entropy is a Simple Measure of the Antibody Profile and is an Indicator of Health Status: A Proof of Concept

**DOI:** 10.1038/s41598-019-54235-6

**Published:** 2019-11-22

**Authors:** Lu Wang, Kurt Whittemore, Stephen Albert Johnston, Phillip Stafford

**Affiliations:** 1 Center for Innovations in Medicine, Biodesign Institute, Arizona State University, Tempe, AZ 85287 United States; 2 Centro Nacional de Investigaciones Oncologicas, Madrid, 28029 Spain

Correction to: *Scientific Reports* 10.1038/s41598-017-18469-6, published online 22 December 2017

The original version of this Article contained errors in the spelling of the authors Kurt Whittemore, Stephen Albert Johnston, & Phillip Stafford which were incorrectly given as K. Whittemore, S. A. Johnston & P. Stafford respectively.

These errors have now been corrected in the PDF and HTML versions of the Article, and in the accompanying Supplementary Material.

