# Supplementary material for: Entropy is a Simple Measure of the Antibody Profile and is an Indicator of Health Status: A Proof of Concept
Source: Sci Rep. 2017 Dec 22;7:18060. doi: 10.1038/s41598-017-18469-6 (PMC5741721; doi:10.1038/s41598-017-18469-6)
Supplement: Supplementary file 1 — Supplemental information [file 41598_2017_18469_MOESM1_ESM.pdf]

Entropy is a Simple Measure of the Antibody Profile and is an Indicator of Health Status

Au:

[1]Wang, Lu

[2]Whittemore, Kurt

[1]Johnston, Stephen Albert

[1]Stafford, Phillip

[1]Center for Innovations in Medicine, Biodesign Institute, Arizona State University, Tempe, AZ 85287

[2]Centro Nacional de Investigaciones Oncologicas, Madrid, Spain, 28029

Supplemental information:

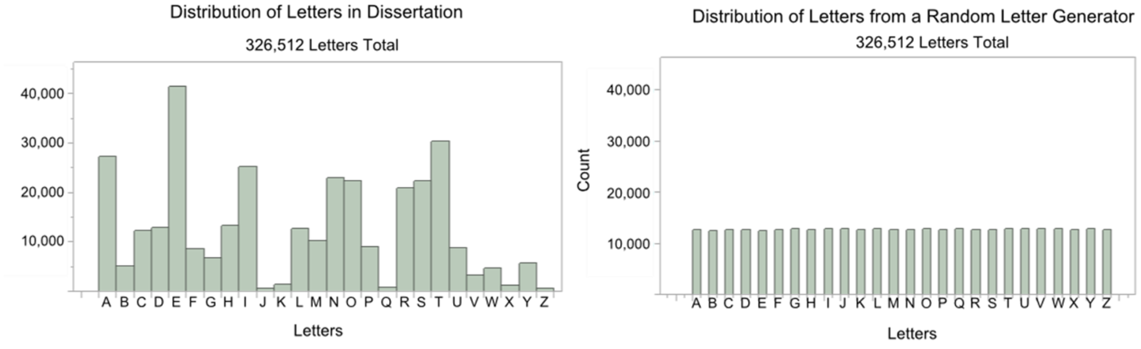

Figure S1. Example of entropy measuring the difference in an information distribution. (a) is the letter distribution from a real dissertation[44]. (b) is the letter distribution of randomly generated thesis with the same total number of letters. The selective use of words results in order for the distribution. The outcome is that the normalized entropy is lower in the real dissertation than the randomly generated one, 0.887 compared with 1.

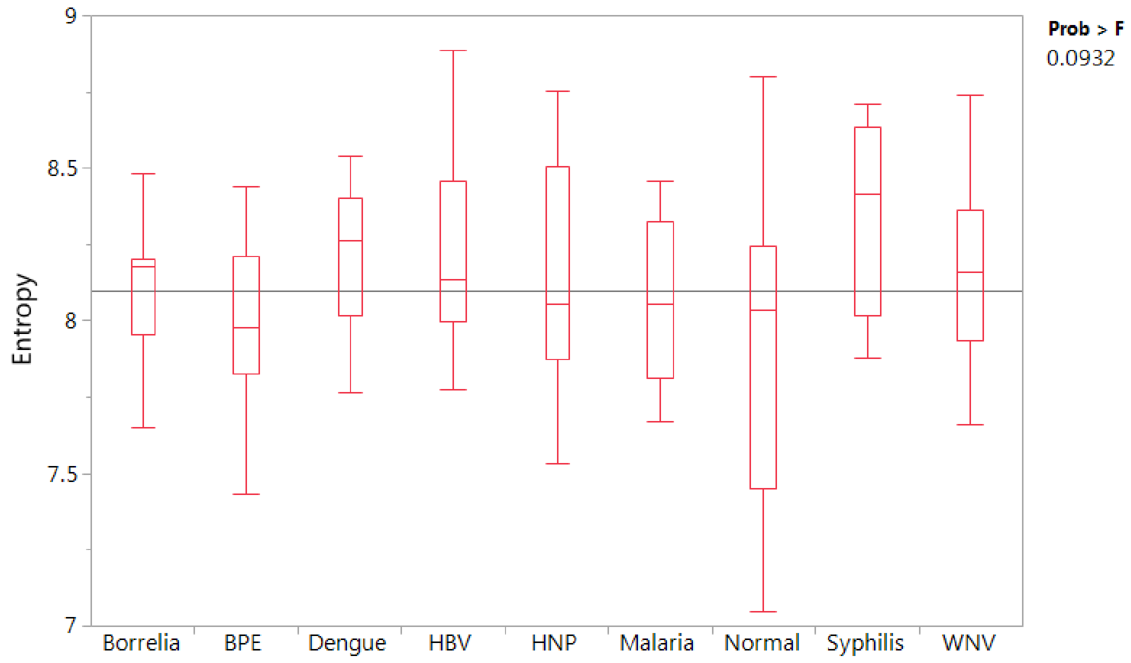

Figure S2. Infections listed individually and in comparison with normal donors. The overall p-value from ANOVA test is not significant (0.0576) from this comparison, nor is the ChiSq probability (0.1862). 6 of the 7 infections have higher mean entropy than normal donors. The horizontal line is the mean of all samples.

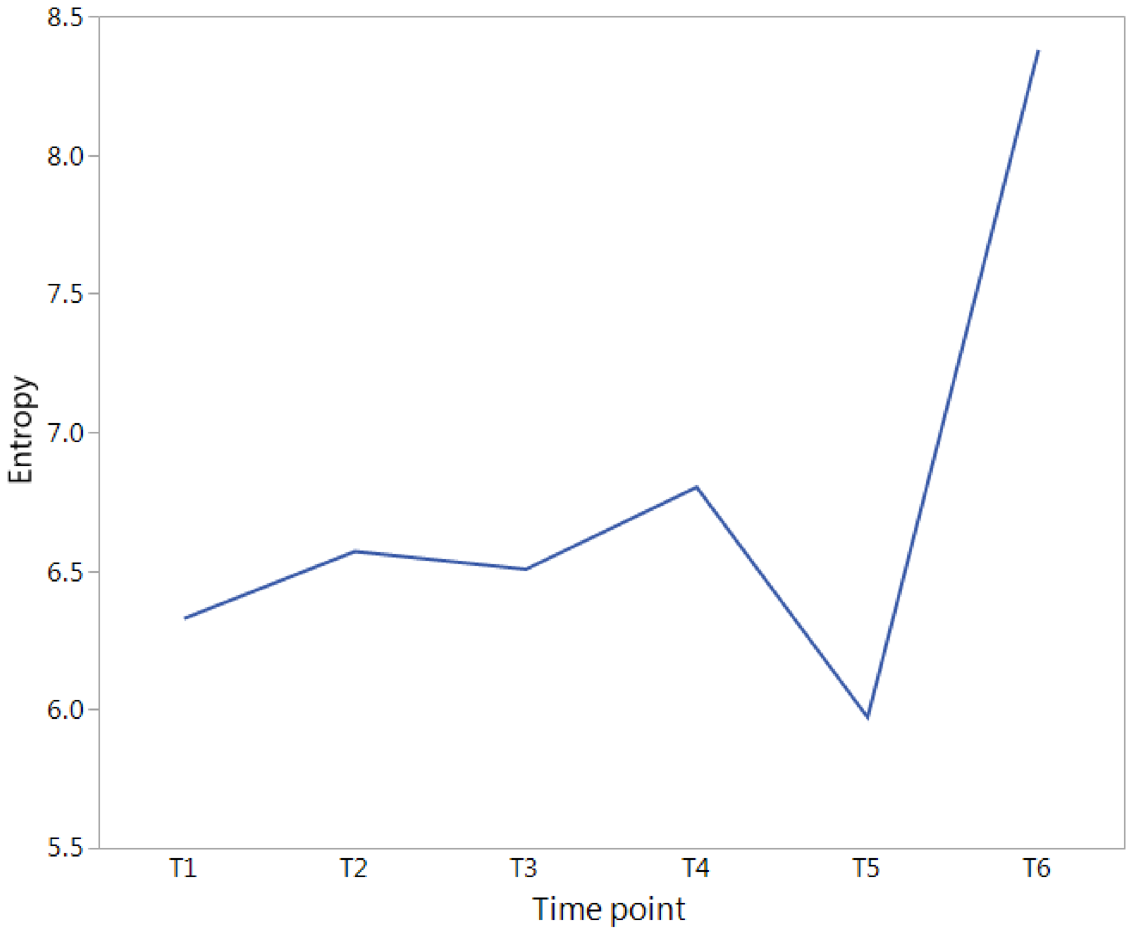

Figure S3. Entropy record of one individual at different time points. The volunteer is healthy at the first 5 data points but report unknown illness at T6. Dramatic increase is observed at T6.

Java ALGORITHM CLASS: This program computes entropy, min, max, variance, standard deviation, Coefficient of Variance (CV), 95th percentile, 5th percentile, and dynamic range from .gpr files (GenePix Report files). The program can be modified to use any microarray dataset by changing the parameters that search for the numeric values within the .gpr file. The jar file below can be run with command line parameters. There are basically six uses of this program:

-find_summary_numbers_one_gpr

-find_summary_numbers_from_folder_of_gprs

-find_summary_numbers_from_tabdelimitedtext_raw_data

-find_summary_numbers_from_tabdelimitedtext_normalized_data

-collectAllSummaryFilesIntoOneTable

-placeFilesInFolderIntoTheirOwnFolder

The bin-size parameter should be set to 1.

Examples below show how to extract from a single .gpr file (1), a folder of .gpr files (2) and a tab delimited text file (3):

1) java -jar "entropy.jar" find_summary_numbers_one_gpr "GPRFILE.gpr" "F532 Median" 5
2) java -jar "entropy.jar" find_summary_numbers_from_folder_of_gprs "GPRFOLDER" "F532 Median" 5
3) java -jar "entropy.jar" find_summary_numbers_from_tabdelimitedtext_raw_data "TABDELIMITEDTEXTFILE.txt" 0 1 2 1 2 1 5

```
import java.util.ArrayList;
import java.util.Arrays
public class SummaryNumberCodeLean {
        UsefulTools useful_tools = new UsefulTools();
        int[] numbers;
        int[] values;
        double[] dnumbers;
        int size = 0;
        long sum = 0;
        double entropy = 0;
        double normalized_entropy = 0;
        double cv = 0;
        double stdev = 0;
        double mean = 0;
        double median = 0;
        int min = 0;
        int max = 0;
        double dmin = 0;
        double dmax = 0;
        double kurtosis = 0;
        double skew = 0;
        double ninety_fifth_percentile = 0;
        double fifth_percentile = 0;
        double dynamic_range = 0;
        double nonn_entropy = 0;
        double nonn_normalized_entropy = 0;
        double nonn_cv = 0;
        double nonn_stdev = 0;
        double nonn_mean = 0;
        double nonn_median = 0;
        double nonn_min = 0;
        double nonn_max = 0;
        double nonn_ninety_fifth_percentile = 0;
        double nonn_fifth_percentile = 0;
        double nonn_dynamic_range = 0;
        double n_mean = 0;
        double n_stdev =0;
        double n_min = 0;
```

```
        double n_max = 0;
        double n_ninety_fifth_percentile = 0;
        double n_fifth_percentile =0;
        public static void main(String[] args) {
                SummaryNumberCodeLean sn = new SummaryNumberCodeLean();
                System.out.println(sn.getTime());
                sn.TestEntropyCalculation_100513d1153();
                System.out.println(sn.getTime());}
        public void TestEntropyCalculation_100513d1103()
        {int[] iarray = {3,5,10,12,5,5};
                System.out.println(calculateEntropy(iarray));}
        public void TestEntropyCalculation_100513d1153()
        {calculateEntropy(iarray);
                calculateSTDEV(iarray);
                cv = stdev/mean;
                median = calculateMedian(iarray);
                kurtosis = useful_tools.kurtosis(iarray, mean, stdev);
                skew = useful_tools.Skew(iarray, mean, stdev);
                ninety_fifth_percentile = useful_tools.percentile(values, 0.95);
                fifth_percentile = useful_tools.percentile(values, 0.05);
                dynamic_range = ninety_fifth_percentile/fifth_percentile;
                nonn_entropy = entropy;
                nonn_normalized_entropy = normalized_entropy;
                nonn_cv = cv;
                nonn_stdev = stdev;
                nonn_mean = mean;
                nonn_median = median;
                nonn_min = min;
                nonn_max = max;
                nonn_ninety_fifth_percentile = ninety_fifth_percentile;
                nonn_fifth_percentile = fifth_percentile;
                nonn_dynamic_range = dynamic_range;
                double[] normalized_array = median_normalize(iarray, median);
                n_mean = getMean(normalized_array);
                getMinAndMax(normalized_array);
                n_min = dmin;
                n_max = dmax;
                n_stdev = calculateSTDEV(normalized_array, n_mean);
                n_ninety_fifth_percentile = useful_tools.percentile(normalized_array,
0.95);
                n_fifth_percentile=useful_tools.percentile(normalized_array, 0.05);
        iarray=multiply_by_factor_and_convert_to_int(normalized_array,10000);
                calculateEntropy(iarray);
                outputValues();}
        public void getSummaryNumbers(int[] iarray)
        {calculateEntropy(iarray);
                calculateSTDEV(iarray);
                cv = stdev/mean;
                median = calculateMedian(iarray);
                kurtosis = useful_tools.kurtosis(iarray, mean, stdev);
                skew = useful_tools.Skew(iarray, mean, stdev);
                ninety_fifth_percentile = useful_tools.percentile(values, 0.95);
                fifth_percentile = useful_tools.percentile(values, 0.05);
                dynamic_range = ninety_fifth_percentile/fifth_percentile;
                nonn_entropy = entropy;
       nonn_normalized_entropy = normalized_entropy;
                nonn_cv = cv;
                nonn_stdev = stdev;
                nonn_mean = mean;
                nonn_median = median;
                nonn_min = min;
                nonn_max = max;
                nonn_ninety_fifth_percentile = ninety_fifth_percentile;
                nonn_fifth_percentile = fifth_percentile;
                nonn_dynamic_range = dynamic_range;
                double[] normalized_array = median_normalize(iarray, median);
                n_mean = getMean(normalized_array);
                getMinAndMax(normalized_array);
                n_min = dmin;
                n_max = dmax;
                n_stdev = calculateSTDEV(normalized_array, n_mean);
```

```
                n_ninety_fifth_percentile = useful_tools.percentile(normalized_array,
0.95);          n_fifth_percentile = useful_tools.percentile(normalized_array, 0.05);
        iarray=multiply_by_factor_and_convert_to_int(normalized_array,10000);
        calculateEntropy(iarray);}
        public void getSummaryNumbers(int[] iarray, int bin_size)
        {calculateEntropy(iarray,bin_size);
                calculateSTDEV(iarray);
                cv = stdev/mean;
                median = calculateMedian(iarray);
                kurtosis = useful_tools.kurtosis(iarray, mean, stdev);
                skew = useful_tools.Skew(iarray, mean, stdev);
                ninety_fifth_percentile = useful_tools.percentile(values, 0.95);
                fifth_percentile = useful_tools.percentile(values, 0.05);
                dynamic_range = ninety_fifth_percentile/fifth_percentile;
                nonn_entropy = entropy;
                nonn_normalized_entropy = normalized_entropy;
                nonn_cv = cv;
                nonn_stdev = stdev;
                nonn_mean = mean;
                nonn_median = median;
                nonn_min = min;
                nonn_max = max;
                nonn_ninety_fifth_percentile = ninety_fifth_percentile;
                nonn_fifth_percentile = fifth_percentile;
                nonn_dynamic_range = dynamic_range;
                double[] normalized_array = median_normalize(iarray, median);
                n_mean = getMean(normalized_array);
                getMinAndMax(normalized_array);
                n_min = dmin;
                n_max = dmax;
                n_stdev = calculateSTDEV(normalized_array, n_mean);
        n_ninety_fifth_percentile = useful_tools.percentile(normalized_array, 0.95);
                n_fifth_percentile = useful_tools.percentile(normalized_array, 0.05);
        iarray=multiply_by_factor_and_convert_to_int(normalized_array,10000);
                calculateEntropy(iarray);}
        public double calculateEntropy(int[] numbers)
        {this.numbers = numbers;
                size = numbers.length;
                values = new int[size];
                max = numbers[0]; //not necessary for entropy calculation
                min = numbers[0];
                for(int i=0; i<size; i++)
                {sum+=numbers[i]; //for the calculation of the mean
                        values[i] = numbers[i];
                        if(numbers[i]<min)
                        {min = numbers[i];}
                        if(numbers[i]>max)
                        {max = numbers[i];}}
                int[] counts = new int[max+1];
                for(int i=0; i<size; i++)
                {try{counts[numbers[i]]++;}
                        catch(Exception e)
                        {int k=0;}}
                double running_sum = 0;
                for(int i=0; i<=max; i++)
                {int value = counts[i];
                        if(value!=0)
                        {double ratio = ((double)value)/size;
                                running_sum+=ratio*Math.log(ratio);}}
                entropy = -running_sum;
                normalized_entropy = entropy/Math.log(size);
                mean = ((double)sum)/size;
                return entropy;}public double calculateEntropy(int[] numbers, double
bin_size)
        {this.numbers = numbers;
                size = numbers.length;
                values = new int[size];
                max = numbers[0]; //not necessary for entropy calculation
                min = numbers[0];
                for(int i=0; i<size; i++)
                {sum+=numbers[i]; //for the calculation of the mean
```

```
                        values[i] = numbers[i];
                if(numbers[i]<min) //not necessary for entropy calculation
                        {min = numbers[i]; //not necessary for entropy calculation
                        }if(numbers[i]>max) //not necessary for entropy calculation
                        {max = numbers[i];}}
                int[] counts = new
int[Double.valueOf(Math.ceil((max+1)/bin_size)).intValue()];
                for(int i=0; i<size; i++)
                {try
                        {int current_number = numbers[i];
                                int current_bin =
Double.valueOf(Math.ceil(current_number/bin_size)).intValue();
                                counts[current_bin-1]++;}
                        catch(Exception e)
                        {int k=0;}}
                double running_sum = 0;
                for(int i=0; i<counts.length; i++)
                {int value = counts[i];
                        if(value!=0)
                        {double ratio = ((double)value)/size;
                        running_sum+=ratio*Math.log(ratio);}}
                entropy = -running_sum;
                normalized_entropy = entropy/Math.log(size);
                mean = ((double)sum)/size;
                return entropy;}
       public double calculateSTDEV(int[] numbers)
    {double sum = 0.0;
        for (int i = 0; i <size; i++) {sum += (numbers[i] - mean) * (numbers[i] - mean);}
        stdev = Math.sqrt(sum / (size - 1));
        return stdev;}
public double calculateSTDEV(double[] numbers, double mean)
    {double sum = 0.0;
        for (int i = 0; i <size; i++) {sum += (numbers[i] - mean) * (numbers[i] - mean);}
        stdev = Math.sqrt(sum / (size - 1));
        return stdev;}public String getTime()
        {return useful_tools.getTime();}
        public double calculateMedian(int[] numbers)
        {Arrays.sort(values);
if (size % 2 == 1){median = values[(size+1)/2-1];}
                else{double lower = values[size/2-1];
                double upper = values[size/2];
                median = (lower + upper) / 2.0;}
                return median;}
                public double[] median_normalize(int[] array, double median)
        {double[] return_list = new double[array.length];
                for(int i=0; i<array.length; i++)
                {return_list[i]=(double)array[i]/median;}
                return return_list;}
        public int[] multiply_by_factor_and_convert_to_int(double[] darray, int factor)
        {int[] return_array = new int[darray.length];
                for(int i=0; i<darray.length; i++)
                {return_array[i] = Double.valueOf(darray[i]*factor).intValue();}
                return return_array;}
        public double getMean(double[] values)
    {double sum = 0.0;
        for(int i=0; i<values.length; i++)
        {sum+=values[i];}
        return sum/(double)values.length;}
                public void getMinAndMax(double[] values)
        {dmin = values[0];
                dmax = values[0];
                for(int i=0; i<values.length; i++)
                {if(values[i]<dmin)
                        {dmin = values[i];}
        if(values[i]>dmax)
                        {dmax = values[i];}}}
        public void outputValues()
        {System.out.println("Entropy: " +nonn_entropy+"\r\n");
                System.out.println("Normalized Entropy: "
+nonn_normalized_entropy+"\r\n");
                System.out.println("Mean: " + nonn_mean);
```

```
                System.out.println("STDEV: " + nonn_stdev);
                System.out.println("CV: " + nonn_cv);
                System.out.println("Median: " + nonn_median);
                System.out.println("Min: " + nonn_min);
                System.out.println("Max: " + nonn_max);
                System.out.println("Kurtosis: " + kurtosis);
                System.out.println("Skew: " + skew);
                System.out.println("Ninetey_fifth_percentile: " +
nonn_ninety_fifth_percentile);
                System.out.println("Fifth_percentile: " + nonn_fifth_percentile);
                System.out.println("Dynamic_range: "+ dynamic_range);
                System.out.println("Entropy_Normalized_Data: " + entropy);
                System.out.println("Normalized_Entropy_Normalized_Data: " +
normalized_entropy);
                System.out.println("Mean_normalized: " + n_mean);
                System.out.println("STDEV_normalized: " + n_stdev);
                System.out.println("Min_normalized: "+ n_min);
                System.out.println("Max_normalized: " + n_max);
                System.out.println("Ninetey_fifth_percentile_normalized: "
+n_ninety_fifth_percentile);
                System.out.println("Fifth_percentile_normalized: " + n_fifth_percentile);}
        public double getEntropy()
        {return nonn_entropy;}
        public double getNormalizedEntropy()
        {return nonn_normalized_entropy;}
        public double getMax()
        {return nonn_max;}
        public double getMin()
        {return nonn_min;}
        public double getCV()
        {return nonn_cv;}
        public double getSTDEV()
        {return nonn_stdev;}
        public double getMean()
        {return nonn_mean;}
        public double getMedian()
        {return nonn_median;}
        public double getFifthPercentile()
        {return nonn_fifth_percentile;}
        public double getNinetyFifthPercentile()
        {return nonn_ninety_fifth_percentile;}
        public double getEntropyNormalizedData()
        {return entropy;}
        public double getNormalizedEntropyNormalizedData()
        {return normalized_entropy;}
        public double getMaxNormalized()
        {return n_max;}
        public double getMinNormalized()
        {return n_min;}
        public double getSTDEVNormalized()
        {return n_stdev;}
        public double getMeanNormalized()
        {return n_mean;}
        public double getFifthPercentileNormalized()
        {return n_fifth_percentile;}
        public double getNinetyFifthPercentileNormalized()
        {return n_ninety_fifth_percentile;}
        public double getKurtosis()
        {return kurtosis;}
        public double getSkew()
        {return skew;}
        public double getDynamicRange()
        {return dynamic_range;}
        public long getSum()
        {return sum;}}
```

Java MAIN (TEST) CLASS

```
import java.io.File;
import java.nio.file.Paths;
```

```
import java.text.DateFormat;
import java.text.SimpleDateFormat;
import java.util.*;
import java.util.regex.Pattern;
public class Test_Immunosignature_Data_030413d0955 {
	private UsefulTools useful_tools = new UsefulTools();
	private DataPreparationClass dpc = new DataPreparationClass();
	private NormalizedDataHandler ndh = new NormalizedDataHandler();
	private CalculationHandler ch = new CalculationHandler();
	TestHandler test_handler = new TestHandler();
	ScenarioHandler sh = new ScenarioHandler();
	String gpr_data_directory = "";
	String result_data_directory = "";
	//set dye_type to "F555 Median" or "F647 Median"
	String dye_type = "F647 Median";
	/**
	 * @param args
	 */
	public static void main(String[] args) {
		if(args.length>0)
		{Test_Immunosignature_Data_030413d0955 tid = new
Test_Immunosignature_Data_030413d0955(args);}
		else{Test_Immunosignature_Data_030413d0955 tid = new
Test_Immunosignature_Data_030413d0955();
			tid.test();	}}
	public Test_Immunosignature_Data_030413d0955()
	public Test_Immunosignature_Data_030413d0955(String[] args)
	{Test_Immunosignature_Data_030413d0955FromCommandLine(args);}
	public Test_Immunosignature_Data_030413d0955(String directory, String filename)
	{System.out.println(useful_tools.getTime());
		ScenarioHandler sh = new ScenarioHandler();
		sh.find_summary_numbers_one_gpr(directory, filename, "F532 Median");
		System.out.println(useful_tools.getTime());}
	public void Test_Immunosignature_Data_030413d0955FromCommandLine(String[] args)
	{/*
		 *
		 * //-collectAllSummaryFilesIntoOneTable(String directory, String
name_of_summary_file, String output_file_name)
	 //--command line version: collectAllSummaryFilesIntoOneTable directory
name_of_summary_file output_file_name
		 *
		 *
		 *
		*/
		String return_string = useful_tools.getTime()+"\r\n";
		String directory = args[1];
		String call_details ="";
		if(args[0].equals("find_summary_numbers_one_gpr"))
		{call_details = "find_summary_numbers_one_gpr_"+args[2];
			if(args[4]!=null)
			{sh.find_summary_numbers_one_gpr(args[1], args[2], args[3],
Integer.valueOf(args[4]).intValue());}else
			{sh.find_summary_numbers_one_gpr(args[1], args[2], args[3]);}
		else if(args[0].equals("find_summary_numbers_from_folder_of_gprs"))
		{call_details = "find_summary_numbers_from_folder_of_gprs_"+args[1];
			if(args[3]!=null)
			{sh.find_summary_numbers_from_folder_of_gprs(args[1], args[2],
Integer.valueOf(args[3]).intValue());}else
			{sh.find_summary_numbers_from_folder_of_gprs(args[1], args[2]);}}
else if (args[0].equals("find_summary_numbers_from_tabdelimitedtext_raw_data"))
		{call_details =
"find_summary_numbers_from_tabdelimitedtext_raw_data_"+args[1];
			if(args[9]!=null)
		{sh.find_summary_numbers_from_tabdelimitedtext_raw_data(args[1], args[2],
Integer.valueOf(args[3]).intValue(), Integer.valueOf(args[4]).intValue(),
Integer.valueOf(args[5]).intValue(), Integer.valueOf(args[6]).intValue(),
Integer.valueOf(args[7]).intValue(), Integer.valueOf(args[8]).intValue(),
Integer.valueOf(args[9]).intValue());}else{
	sh.find_summary_numbers_from_tabdelimitedtext_raw_data(args[1], args[2],
Integer.valueOf(args[3]).intValue(), Integer.valueOf(args[4]).intValue(),
Integer.valueOf(args[5]).intValue(), Integer.valueOf(args[6]).intValue(),
```

```
Integer.valueOf(args[7]).intValue(), Integer.valueOf(args[8]).intValue());}}
               else
if(args[0].equals("find_summary_numbers_from_tabdelimitedtext_normalized_data")
               {call_details =
"find_summary_numbers_from_tabdelimitedtext_normalized_data_"+directory;
       if(args[9]!=null)
       {sh.find_summary_numbers_from_tabdelimitedtext_normalized_data(args[1], args[2],
Integer.valueOf(args[3]).intValue(), Integer.valueOf(args[4]).intValue(),
Integer.valueOf(args[5]).intValue(), Integer.valueOf(args[6]).intValue(),
Integer.valueOf(args[7]).intValue(), Integer.valueOf(args[8]).intValue(),
Integer.valueOf(args[9]).intValue());}else
       {sh.find_summary_numbers_from_tabdelimitedtext_normalized_data(args[1], args[2],
Integer.valueOf(args[3]).intValue(), Integer.valueOf(args[4]).intValue(),
Integer.valueOf(args[5]).intValue(), Integer.valueOf(args[6]).intValue(),
Integer.valueOf(args[7]).intValue(), Integer.valueOf(args[8]).intValue());}}
               else if(args[0].equals("collectAllSummaryFilesIntoOneTable"))
               {call_details = "collectAllSummaryFilesIntoOneTable_"+directory;
                       sh.collectAllSummaryFilesIntoOneTable(args[1], args[2], args[3]);}
               else if(args[0].equals("placeFilesInFolderIntoTheirOwnFolder"))
               {call_details = "placeFilesInFolderIntoTheirOwnFolder_"+directory;
               sh.placeFilesInFolderIntoTheirOwnFolder(args[1]);}
               return_string+=useful_tools.getTime();
               call_details=call_details.replaceAll(":", "C");
               call_details=call_details.replaceAll(Pattern.quote(File.separator),
"S");useful_tools.createTextFile(directory, "runtime_info.txt", return_string);}
       public void test()
       {System.out.println(useful_tools.getTime());
               TestHandler th = new TestHandler();
       {"find_summary_numbers_from_tabdelimitedtext_normalized_data","};
       {"find_summary_numbers_from_tabdelimitedtext_normalized_data","YourDirectoryHere",
"2","1","2","1","2","6"};
String[] arguments = {"find_summary_numbers_one_gpr", "YourDirectoryHere","4-46 S2 F1 Hi
P20 110512 ND145 50k S","F532 Median", "5"};
Test_Immunosignature_Data_030413d0955FromCommandLine(arguments);
{"find_summary_numbers_from_folder_of_gprs","YourDirectoryHere","F532
Median","true","1","20","10"};
"YourDirectoryHere", "F647 Median", "false", "1", "65535", "10000"};
System.out.println(useful_tools.getTime());}}
```
